# 1558. Associations between gender identity and solicited adverse events after passive infusion of VRC01 or placebo in HVTN 704/HPTN 085

**DOI:** 10.1093/ofid/ofad500.1393

**Published:** 2023-11-27

**Authors:** Deborah Theodore, Moni Neradilek, Kevin Gillespie, Srilatha Edupuganti, Juan Carlos Hinojosa, Javier R Lama, Robert De La Grecca, Annet Davis, Daniel J Mangini, Philip Andrew, Mary Marovich, Sheryl Zwerski, Delivette Castor, Alison C Roxby, Myron Cohen, Lawrence Corey, Yunda Huang, Shelly Karuna, Magdalena E Sobieszczyk

**Affiliations:** Columbia University Irving Medical Center, New York, New York; SCHARP (Fred Hutch Cancer Center), Seattle, Washington; SCHARP (Fred Hutch), Seattle, Washington; Emory University School of Medicine, Decatur, GA; Asociacion Civil Selva Amazonica, Iquitos, Loreto, Peru; Asociacion Civil Impacta Salud y Educacion, Lima, Lima, Peru; HIV Vaccine Trials Network (HVTN) at Fred Hutchinson Cancer Research Center, Lima, Lima, Peru; University of Pennsylvania, Philadelphia, Pennsylvania; University of Pennsylvania, Philadelphia, Pennsylvania; FHI 360, HIV Prevention Trials Network, Durham, NC; National Institute of Allergy and Infectious Diseases, National Institutes of Health, Bethesda, Maryland; National Institutes of Health, Rockville, Maryland; Columbia University Medical Center, New York, New York; University of Washington, Seattle, Washington; University of North Carolina at Chapel Hill, Chapel Hill, North Carolina; Fred Hutchinson Cancer Research Center, Seattle, WA; Fred Hutch, Seattle, Washington; GreenLight Biosciences & Fred Hutch Cancer Center, Seattle, Washington; Division of Infectious Diseases, Department of Medicine, Vagelos College of Physicians and Surgeons, New York-Presbyterian Columbia University Irving Medical Center, New York, NY, USA, New York, New York

## Abstract

**Background:**

Gender minority individuals are understudied in clinical trials. Realizing the potential of HIV prevention options requires understanding product tolerability across diverse groups vulnerable to HIV acquisition. Within HVTN 704/HPTN 085, a phase 2b trial of the broadly neutralizing antibody (bnAb) VRC01 for HIV prevention, analyses of associations between gender identity and solicited adverse events (solAE) after infusions of VRC01 or saline placebo have not been reported.

**Methods:**

HVTN 704/HPTN 085 enrolled men who have sex with men and transgender (TG) participants from Brazil, Peru, Switzerland and the US and randomized them 1:1:1 to receive an infusion every 8 weeks (10 total) of VRC01 30 mg/kg, VRC01 10 mg/kg or placebo. solAE were recorded for 3 days after each infusion. Gender was defined by self-report and sex assigned at birth. Odds ratios of the association of gender (cisgender men [CM] vs. gender minority participants [including TG women, TG men or other identity]) and frequency and severity of solAE were adjusted for age, race and ethnicity.

**Results:**

Of 2,552 participants, 162 identified as TG women (6.3%), 75 as other identity (2.9%) and 15 as TG men (0.6%). Gender minority participants identified as Black/African American (8.7%), other (59.9%) and White (21.8%); 68.3% identified as Hispanic/Latino (**Tab. 1**). Overall, 867 (37.7%) CM and 107 (42.5%) gender minority participants reported a solAE (**Tab. 2**). Gender minority participants were more likely to report solAE than CM overall (OR 1.59, 95% CI 1.21-2.10, p=0.001) and among placebo recipients (1.72, 1.05-2.81, 0.032) (**Tab. 3**). SolAE severity (≥Grade 2) did not significantly differ overall (1.83, 0.79-4.20, 0.174). Grade 2 solAE were reported after 1% and 2% of infusions among CM and gender minority participants, respectively. Grade 3 and 4 events were rare in both groups (< 0.1%). Infusion completion did not differ.

**Table 1.**
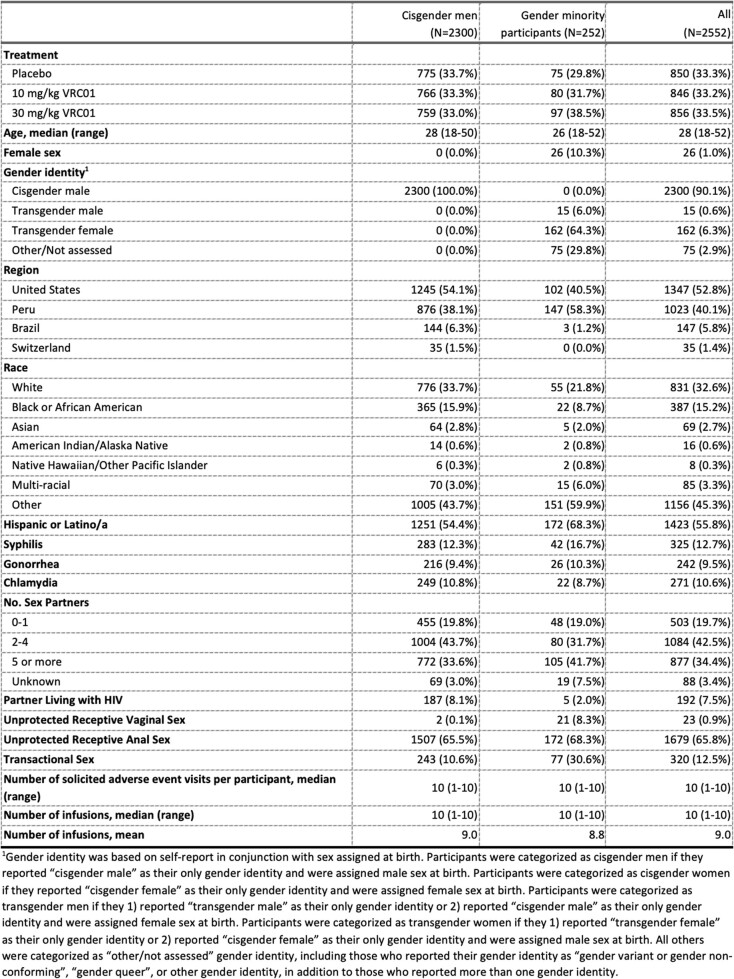
Demographic and behavioral characteristics by gender identity of participants in HVTN 704/HPTN 085 who did not acquire HIV during the trial.

**Table 2.**
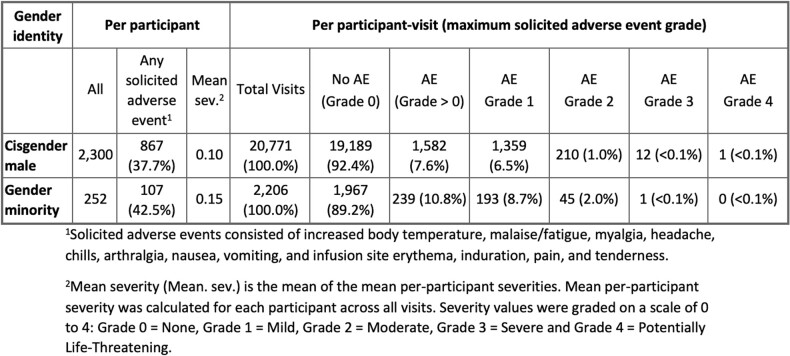
Frequency and severity of solicited adverse events by gender identity among VRC01 and placebo recipients.

**Table 3.**
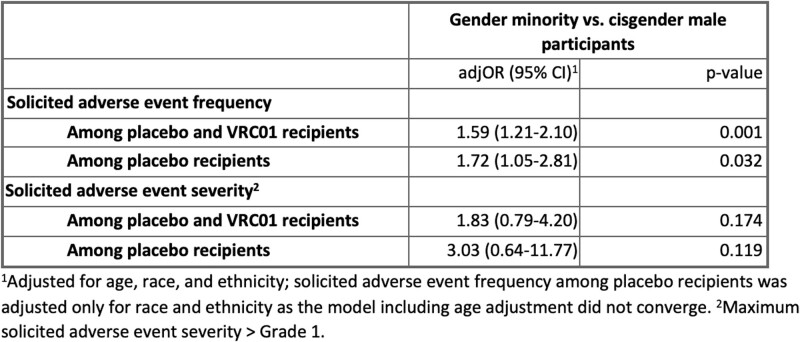
Adjusted effect of gender identity on solicited adverse event frequency and severity.

**Conclusion:**

HVTN 704/HPTN 085 is the first large-scale HIV bnAb preventive trial allowing assessment of associations between gender identity and solAE. Gender minority participants had more frequent solAE, but importantly, infusion completion did not differ and severe solAEs were rare. HIV prevention and bnAb trials must engage and include gender minority individuals to evaluate the tolerability of novel agents.

**Disclosures:**

**Srilatha Edupuganti, MD MPH FIDSA**, Sanofi: Grant/Research Support

